# A Two Edged Sword

**Published:** 1885-12

**Authors:** 


### A TWO-EDGED SWORD.

There is a law in force in New York which will punish you if you don't, and punish you if you do. It requires physicians, under penalty, to report the existence of dangerous diseases in their practice. Dr. Purdy reported a millinery woman with small pox, and she was taken to the small-pox hospital. His diagnosis was confirmed by the Health Officer. It was a light case, and the woman, recovering in a short time, denied that it was small pox, and laid it to the use of cosmetics containing acetic acid. She sued the Doctor for $10,000 damages for breaking up her business—saying the *report* kept her customers from coming near her. The Court gave her a verdict for $500. The case will be appealed, and the Doctor has asked his State Association to come to his assistance and help him out. Another argument for organization of the medical profession for the protection of its members.

This is very much like the situation of the little boy whose mother said, threateningly, "Johny, if you go to that baseball play, I'll make you *wish you hadn't.*" "And if I don't go I'll allers wish I had," said Johny, "so I believe I'm off anyhow." Or like the Dutchman who whipped his sullen son because he looked like he thought "damn it." We reckon Dr. Purdy thinks "d—— it."

